# Phenotypic Spectrum and Prognosis of Epilepsy Patients With *GABRG2* Variants

**DOI:** 10.3389/fnmol.2022.809163

**Published:** 2022-03-14

**Authors:** Ying Yang, Xueyang Niu, Miaomiao Cheng, Qi Zeng, Jie Deng, Xiaojuan Tian, Yi Wang, Jing Yu, Wenli Shi, Wenjuan Wu, Jiehui Ma, Yufen Li, Xiaoling Yang, Xiaoli Zhang, Tianming Jia, Zhixian Yang, Jianxiang Liao, Yan Sun, Hong Zheng, Suzhen Sun, Dan Sun, Yuwu Jiang, Yuehua Zhang

**Affiliations:** ^1^Department of Pediatrics, Peking University First Hospital, Beijing, China; ^2^Department of Neurology, Shenzhen Children’s Hospital, Shenzhen, China; ^3^Department of Neurology, Beijing Children’s Hospital, Capital Medical University, Beijing, China; ^4^Department of Neurology, National Children’s Medical Center, Children’s Hospital of Fudan University, Shanghai, China; ^5^Department of Neurology, Children’s Hospital of Xinjiang Uygur Autonomous Region, Xinjiang Hospital of Beijing Children’s Hospital, Ürümqi, China; ^6^Department of Pediatrics, The First Affiliated Hospital of Henan University of Chinese Medicine, Zhengzhou, China; ^7^Department of Neurology, Hebei Children’s Hospital, Shijiazhuang, China; ^8^Department of Neurology, Wuhan Children’s Hospital, Tongji Medical College, Huazhong University of Science and Technology, Wuhan, China; ^9^Department of Pediatrics, Linyi People’s Hospital, Linyi, China; ^10^Department of Pediatrics, The Third Affiliated Hospital of Zhengzhou University, Zhengzhou, China

**Keywords:** GABRG2, epilepsy, infancy, fever-sensitive, prognosis

## Abstract

**Objective:**

This study aimed to obtain a comprehensive understanding of the genetic and phenotypic aspects of *GABRG2*-related epilepsy and its prognosis and to explore the potential prospects for personalized medicine.

**Methods:**

Through a multicenter collaboration in China, we analyzed the genotype-phenotype correlation and antiseizure medication (ASM) of patients with *GABRG2*-related epilepsy. The three-dimensional protein structure of the *GABRG2* variant was modeled to predict the effect of *GABRG2* missense variants using PyMOL 2.3 software.

**Results:**

In 35 patients with *GABRG2* variants, 22 variants were *de novo*, and 18 variants were novel. The seizure onset age was ranged from 2 days after birth to 34 months (median age: 9 months). The seizure onset age was less than 1 year old in 22 patients (22/35, 62.9%). Seizure types included focal seizures (68.6%), generalized tonic-clonic seizures (60%), myoclonic seizures (14.3%), and absence seizures (11.4%). Other clinical features included fever-sensitive seizures (91.4%), cluster seizures (57.1%), and developmental delay (45.7%). Neuroimaging was abnormal in 2 patients, including dysplasia of the frontotemporal cortex and delayed myelination of white matter. Twelve patients were diagnosed with febrile seizures plus, eleven with epilepsy and developmental delay, two with Dravet syndrome, two with developmental and epileptic encephalopathy, two with focal epilepsy, two with febrile seizures, and four with unclassified epilepsy. The proportions of patients with missense variants in the extracellular region and the transmembrane region exhibiting developmental delay were 40% and 63.2%, respectively. The last follow-up age ranged from 11 months to 17 years. Seizures were controlled in 71.4% of patients, and 92% of their seizures were controlled by valproate and/or levetiracetam.

**Conclusion:**

The clinical features of *GABRG2*-related epilepsy included seizure onset, usually in infancy, and seizures were fever-sensitive. More than half of the patients had cluster seizures. Phenotypes of *GABRG2*-related epilepsy were ranged from mild febrile seizures to severe epileptic encephalopathies. Most patients with *GABRG2* variants who experienced seizures had a good prognosis. Valproate and levetiracetam were effective treatments for most patients.

## Introduction

**FIGURE 1:**
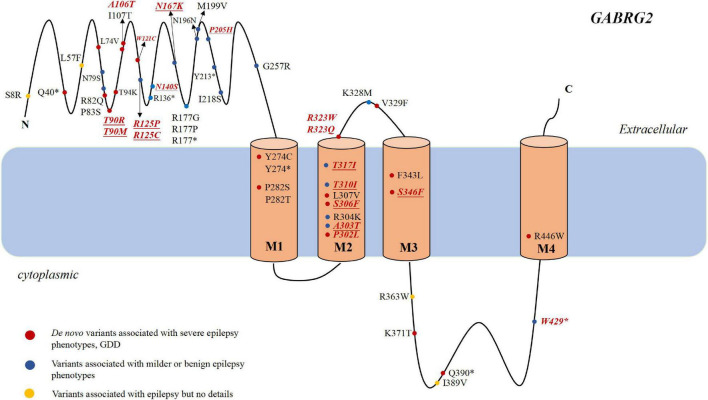
The genotype and phenotype of *GABRG2* pathogenic variants in patients with epilepsy. Representation of the GABA_*A*_ receptor protein structure and the location of *GABRG2* variants previously described in the literature (marked in black) and identified in our cohort (marked in red and italics). The novel variants reported for the first time in the present study are indicated in underscored. The red dots indicate severe phenotypes, including epileptic encephalopathy and developmental delay along with epilepsy. The blue dots indicate mild phenotypes, including febrile seizure plus and GEFS+. The yellow dots indicate the variants associated with epilepsy but no details are available. *Represents the nonsense variant.

Epilepsy is characterized by an enduring predisposition to generate epileptic seizures. Numerous genetic defects underlying different forms of epilepsy have been identified with most of these genes encoding ion channel proteins (Surguchov et al., 2017). *GABRG2* (OMIM:137164) resides on chromosome 5q34 and is a member of the gamma-aminobutyric acid-A (GABA_*A*_) receptor gene family of heteromeric pentameric ligand-gated ion channels. GABA_*A*_ receptor subunits contains a extracellular N-terminus, four transmembrane domains, a small extracellular loop between the second and third transmembrane domains and a larger intracellular loop between the third and the fourth transmembrane domains (Figure 1). The first variant in *GABRG2* (p.R43Q) was reported to be related to genetic epilepsy with febrile seizures (GEFS+) in 2001 in an Australian family (Wallace et al., 2001). In the same year, Baulac et al. (2001) identified a *GABRG2* heterozygous missense variant (K328M) in a 3-generation French family with variable seizure phenotypes and most consistent with GEFS+. With the broad application of next-generation sequencing (NGS) in patients with epilepsy, pathogenic variants of *GABRG2* related to developmental and epileptic encephalopathy 74 (DEE74, OMIM:618396) were reported. To our knowledge, no detailed descriptions of the phenotypic spectrum of patients with epilepsy carrying *GABRG2* variants are available. Therefore, this study aimed to determine the phenotypic spectrum of epilepsy patients carrying *GABRG2* variants and the prognosis of patients in a Chinese cohort from multicenter.

## Materials and Methods

### Participants

Genetic testing was conducted in all patients diagnosed with epilepsy without acquired factors (e.g., perinatal brain injury, traumatic brain injury, and central nervous system infections). Thirty-five patients with epilepsy and *GABRG2* variants who attended the Pediatric Department of Peking University First Hospital from January 2008 to August 2021 were included in this study, including 22 males and 13 females. Clinical information about the age at seizure onset, seizure frequency, seizure types, developmental milestones, neurological status, family history, the results of accessory examinations including electroencephalogram (EEG) and brain magnetic resonance imaging (MRI), and treatment were collected in the clinic. Brain MRI and video EEG were reviewed by neuroradiologists and neurophysiologists, respectively. All patients identified with *GABRG2* variants were followed in outpatient settings or by telephone at least every 3 months.

The Ethics Committee of Peking University First Hospital and the institutional review boards of collaborating groups approved the project (approval number 2012[453]). Written informed consent was obtained from the parents of all patients. The study was performed in accordance with the ethical standards established in the 1964 Declaration of Helsinki and its later amendments.

### Genetic Analysis

*GABRG2* variations (NM_000816, GRCh37/hg19) were determined using targeted next-generation sequencing of epilepsy (epilepsy gene panel) or whole-exome sequencing. Synonymous variants and single nucleotide polymorphisms with minor allele frequencies greater than 5% were removed^1^. Functional consequences were predicted by Mutation Taster^2^, Polyphen-2^3^, and PROVEAN^4^. The pathogenicity of variants was evaluated according to the American College of Medical Genetics and Genomics (ACMG) guidelines (Richards et al., 2015). Sanger sequencing was used to verify variations and identify the inheritance of variants.

### Structural Modeling

Three-dimensional protein structure were modeled to predict the effect of *GABRG2* missense variants using PyMOL 2.3 software, and the pathogenicity of candidate variants was evaluated.

## Results

### Genetic Analysis

Our cohort of 35 patients carried 24 unique *GABRG2* variants, including 27 missense variants, 5 splicing variants, 1 nonsense variant, 1 frameshift variant, and 1 small deletion variant (Table 1 and Supplementary Table 1). Eighteen variants were novel (p.T90M, p.W121C, p.R125P, p.R125C, p.N140S, p.N167K, p.P205H, c.631 + 4A > G, c.631 + 5G > T, c.922 + 1G > T, p.A303T, p.S306F, p.T310I, p.T317I, c.1128 + 5G > A, p.C382Sfs*57, c.1249-7C > T, and exon1-11 deletion). Six variants (p.T90R, p.A106T, p.P302L, p.R323W, p.R323Q and p.W429X) have been previously reported (Sun et al., 2008; Shen et al., 2017; Zou et al., 2017; Hernandez et al., 2021). Variants T310I, A106T, and R323W recurred twice, and R323Q recurred nine times, respectively. Twenty-two patients carried *de novo* variants and 13 patients had inherited variants. None of these variants were found in the Genome Aggregation Database.

**TABLE 1:** The phenotype and genotype of 35 patients with *GABRG2* variants in our cohort.

#	Gender	variants	Inheritance	Family history: Y or N	Seizure-onset age	Seizure types	Seizures fever sensitivity: Y or N	Cluster seizures: Y or N	Developmental	EEG	Brain MRI	Other clinical findings	Diagnosis	AEDs	Seizure-off age	Age at last follow-up
1	F	c.269C > G/p.T90R	*De novo*	N	7 months	FS, GTCS, AS, focal SE	Y	Y	delay	MF	Normal (15 years 8 months)	NS	Dravet syndrome	LEV	15 years 9 months	17 years
2	F	c.269C > T/p.T90M	Maternal	Y	1 year 4 months	FS	Y	N	normal	normal	Normal (5 years 8 months)	NS	Focal epilepsy	VPA; LEV; LTG	8 years	9 years 10 months
3	M	c.316G > A/p.A106T	*De novo*	N	40 days	FS	N	N	delay	GFW	Dysplasia of the frontal and temporal cortex, delayed myelination (1y)	Microcephaly	DEE	VPA; OXC	1 year	3 years 8 months
4	M	c.316G > A/p.A106T	*De novo*	N	2 days	FS, GTCS SE	N	Y	delay	FSS	normal (6 months)	NS	DEE	TPM; LEV	5 month	1 year 11 months
5	M	c.363G > C/p.W121C	*De novo*	N	9 months	GTCS	Y	N	delay	normal	normal (9 months)	NS	Epilepsy and developmental delay	VPA	1 year 2 months	3 years 7 months
6	M	c.374G > C/p.R125P	Maternal	Y	9 months	GTCS, FS	Y	Y	normal	normal	NA	NS	FS +	VPA	2 years 10 months	3 years 10 months
7	F	c.373C > T/p.R125C	Maternal	Y	2 years 1 month	FS	Y	N	normal	FSS	normal (5 years 9 months)	NS	FS +	VPA	5 years 11 months	8 years
8	M	c.419A > G/p.N140S	Maternal	Y	1 year 6 months	GTCS	Y	Y	normal	NA	normal	NS	FS	LEV	3 years 3 months	5 years 7 months
9	M	c.501C > A/p.N167K	*De novo*	N	7 months	GTCS	Y	Y	normal	GSW	NA	NS	FS +	VPA	1 year 9 months	3 years 4 months
10	F	c.614C > A/p.P205H	*De novo*	N	9 months	GTCS	Y	Y	normal	GSW, FSS, DS	Normal (9 months)	NS	FS	LEV	Ongoing	11 months
11	M	c.631 + 4A > G	Maternal	Y	9 months	GTCS	Y	N	normal	normal	normal	NS	FS +	LEV	2 years 3 months	3 years 1 month
12	M	c.631 + 5G > T	Paternal	Y	9 months	GTCS, FS	Y	Y	normal	normal	Normal (2 years)	NS	FS +	VPA; OXC	Ongoing	2 years 2 months
13	M	c.922 + 1G > T	Maternal	Y	2 years	FS	Y	N	normal	FSS	NA	NS	FS +	LEV	3 years	4 years 9 months
14	M	c.905C > T/p.P302L	*De novo*	N	7 months	GTCS, FS, AS, MS, GTCS SE	Y	Y	delay	GSW, MF	normal (10 years)	NS	Dravet syndrome	VPA; *OXC*; CLB	Ongoing	12 years
15	M	c.907G > A/p.A303T	*De novo*	N	2 years	GTCS	Y	N	normal	MF, GSW	normal	NS	Epilepsy	VPA	13 years 8 months	14 years 10 months
16	F	c.917C > T/p.S306F	*De novo*	N	1 year 8 months	FS	N	Y	delay	DS	normal	NS	Epilepsy and developmental delay	NA	Ongoing	9 years
17	M	c.929C > T/p.T310I	*De novo*	N	6 months	FS	Y	Y	normal	GSW	Normal (1 year)	NS	Focal epilepsy	VPA; LEV	4 years 11 months	6 years 7 months
18	F	c.929C > T/p.T310I	*De novo*	N	8 months	GTCS, MS	Y	Y	normal	FSS	Normal (1 year)	ADHD	Epilepsy	VPA; LEV	2 years 2 months	12 years
19	M	c.950C > T/p.T317I	*De novo*	N	8 months	FS	Y	Y	normal	Normal	Normal (1 year 4 months)	NS	FS +	VPA	5 years 4 months	6 years 8 months
20	F	c.967C > T/p.R323W	Maternal	Y	9 months	FS, SE	Y	N	delay	GSW	normal	NS	Epilepsy and developmental delay	VPA	Ongoing	1 year 3 months
21	M	c.967C > T/p.R323W	*De novo*	N	8 months	GTCS, FS	Y	N	delay	Normal	normal	NS	Epilepsy and developmental delay	LEV; CZP; VPA	Ongoing	9 years
22	M	c.968G > A/p.R323Q	Paternal	Y	1 year 3 months	GTCS	Y	N	normal	Normal	normal	NS	FS +	LEV	2 years 3 months	4 years
23	M	c.968G > A/p.R323Q	*De novo*	N	10 months	MS, AS	Y	N	delay	GSW, DS, MF	normal	NS	Epilepsy and developmental delay	VPA; LEV; PER	4 years 4 months	7 years
24	M	c.968G > A/p.R323Q	*De novo*	N	8 months	FS	Y	N	delay	NA	NA	NS	Epilepsy and developmental delay	VPA; LEV	6 years 6 months	8 years
25	M	c.968G > A/p.R323Q	*De novo*	N	8 months	FS	Y	Y	delay	FSS	Dysplasia of the frontal and temporal cortex, delayed myelination (1 year 6 months)	NS	Epilepsy and developmental delay	VPA	1 year	4 years
26	F	c.968G > A/p.R323Q	*De novo*	N	1 year 1 month	GTCS, MS, FS	Y	Y	delay	GSW	normal	NS	Epilepsy and developmental delay	VPA; LEV	2 years 3 months	3 years 3 months
27	F	c.968G > A/p.R323Q	*De novo*	N	1 year 1 month	GTCS, FS	Y	N	delay	Normal	Normal (6 years)	NS	Epilepsy and developmental delay	VPA; LEV	6 years	8 years
28	F	c.968G > A/p.R323Q	*De novo*	N	6 months	FS	Y	Y	delay	FSS	Normal (9 months)	NS	Epilepsy and developmental delay	VPA; LEV	1 year 7 months	4 years
29	F	c.968G > A/p.R323Q	*De novo*	N	11 months	GTCS, FS	Y	Y	delay	DS, MF	Normal (1 year)	NS	Epilepsy and developmental delay	PER; VPA; CZP; *LTG;* OXC	Ongoing	1 year 3 months
30	M	c.968G > A/p.R323Q	*De novo*	N	10 months	GTCS, FS, AS	Y	Y	normal	FSS	Normal (3 years)	NS	Epilepsy	VPA; LEV	Ongoing	3 years 6 months
31	F	c.1128 + 5G > A	*De novo*	N	2 years 3 months	MS, GTCS	Y	Y	normal	GSW	Normal (2 years 6 months)	NS	Epilepsy	VPA; LEV	Ongoing	2 years 10 months
32	F	c.1140_1143del/ p.C382Sfs*57	Maternal	Y	2 years 10 months	GTCS	Y	N	normal	Normal	Normal (3 years)	NS	FS +	VPA	3 years	4 years 4 months
33	M	c.1249-7C > T	Maternal	Y	1 year 6 months	FS	Y	Y	normal	DS, FSS	Normal (3 years)	NS	FS +	VPA; *TPM; CZP; NZP*	3 years 3 months	5 years 10 months
34	M	c.1287G > A/p.W429X	Maternal	Y	1 year 3 months	FS	Y	Y	normal	Normal	Normal	NS	FS +	VPA	6 years 6 months	14 years
35	M	exon 1-11 deletion	Maternal	Y	9 months	GTCS	Y	N	normal	FSS	Normal (1 year 6 months)	NS	FS +	VPA; LEV	Ongoing	2 years 2 months

*F, female; M, male; AS, absence seizure; C, clonic seizure; DEE, developmental and epileptic encephalopathy; FS, focal seizure; GTCS, generalized tonic-clonic seizure; MS, myoclonic seizure; SE, status epilepticus; T, tonic seizure; TC, tonic-clonic seizure; NA, not applicable; NS, no specific. EEG, electroencephalography; DS, diffuse slowing; FSS, focal spike slow waves; MF, multifocal; GSW, generalized spike wave; GFW, generalized fast wave; MRI, magnetic resonance imaging; ASD autistic spectrum disorder; AED, anti-epileptic therapy; LEV, levetiracetam; VPA, valproic acid; TPM, topiramate; CLB, clobazam; VGB, vigabatrin; DZP, diazepam; PB, phenobarbital; PER, Perampanel; CZP, clonazepam; KD, ketogenic diet; OXC, oxcarbazepine. Underlining indicates treatment with clinical response (decreased seizure frequency or severity), and italics indicates a negative response (aggravation of seizure frequency and/or severity).*

### Seizure Types

In 35 children with epilepsy carrying *GABRG2* variants, the seizure onset age ranged from 2 days to 34 months of age (median age: 9 months). The seizure onset age was less than 1 year old in 22 patients (22/35, 62.9%). Seizure types were predominantly focal seizures (24/35, 68.6%). Other seizure types included generalized tonic-clonic seizures (21/35, 60%), myoclonic seizures (5/35, 14.3%), and absence seizures (4/35, 11.4%). A striking feature was the occurrence of fever-sensitive seizures in 32/35 (91.4%) patients. Notably, 57.1% (20/35) patients had a cluster of seizures.

### Neurodevelopment and Additional Comorbidities

In our cohort, 15 patients (42.8%, 15/35) experienced developmental delays. Two patients (patients 3, and 4) were unable to raise their heads at the age of 1 year. One patient (patient 29) walked after the age of 2 years. Five patients (patients 1, 14, 18, 24, and 25) walked at approximately the age of 18 months but had poor motor coordination. Patient 1 expressed herself in simple language with no more than four words at the age of 16 years. Patient 13 had a mild speech impediment, and he spoke disfluently at 6 years of age. Patient 14 also had learning difficulties. Eight patients had a mild intellectual disability (patients 5, 16, 20, 21, 23, 26, 27, and 28), and some of them had learning difficulties in school (patients 16, 21, 23, and 27). Microcephaly was observed in 1 patient (patient 3). One patient (patient 18) was diagnosed with attention deficit and hyperactivity disorder (ADHD). Twenty patients showed normal psychomotor development at the last follow-up.

### Video Electroencephalogram and Brain Imaging

Thirty-five patients underwent video EEG monitoring for 4–24 h (Table 1). The EEG exhibited diffuse slow background activity in 5 patients. Interictal epileptiform discharges were captured in 24 patients, including multifocal spike-slow waves in 5 patients and focal spike-slow waves in 10 patients. Generalized spike-wave or polyspike wave discharges were observed in 9 patients. Seizures were observed in 7 (20%, 7/35) patients, consisting of focal seizures in 3, absence seizures in 3, and myoclonic seizures in one patient. Eleven patients had a normal EEG.

**FIGURE 2:**
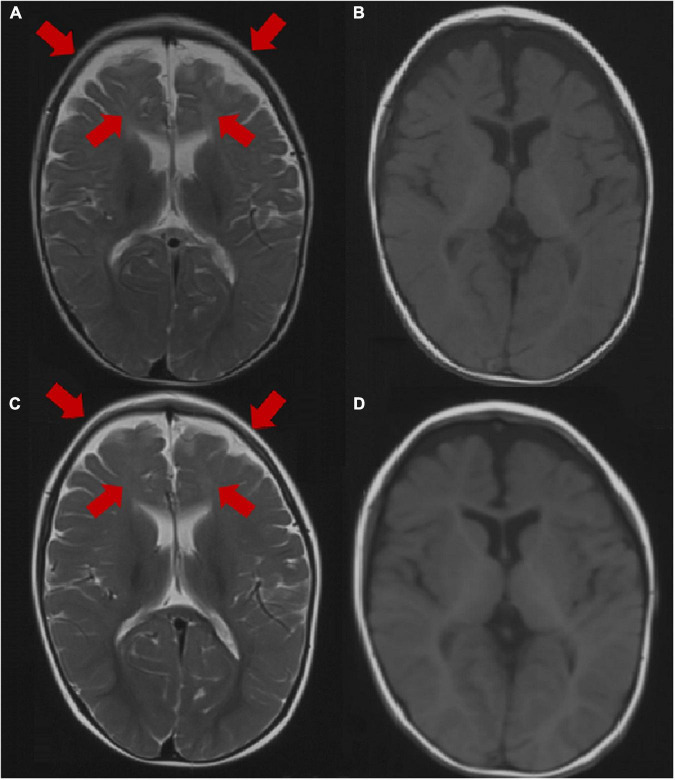
Brain MRI image of patient 3 at the age of 1 year. **(A,B)** Axial images (T1WI and T2WI) showing dysplasia of the frontal and temporal cortex and delayed myelination. Brain MRI image of patient 3 at the age of 2 years. **(C,D)** Axial images (T1WI, T2WI) showing dysplasia of the frontal cortex and delayed myelination. The arrow points to the lesion.

Brain MRI was abnormal in 2 patients (2/31, 6.5%), including dysplasia of the frontotemporal cortex and delayed myelination of white matter in 2 patients (patient 3 and patient 25). The brain MRI of 29 patients was normal at the last follow-up. The brain MRI results of patient 3 are shown in Figure 2.

### Diagnosis of the Phenotype

Twelve of 35 patients were diagnosed with FS+. Eleven patients were diagnosed with developmental delays and epilepsy, and their seizure onset ages ranged from 6 to 20 months. Two patients were diagnosed with Dravet syndrome. They had various seizure types including focal seizures, GTCS, myoclonic seizures, atypical absence seizures, and seizures with fever-sensitivity. Two patients (patients 3 and 4) were diagnosed with DEE, and their seizure onset ages were 2 days and 40 days. Two patients (patients 2 and 17) were diagnosed with focal epilepsy. Two patients (patients 8 and 10) had FS, one of whom inherited the variant from his mother (patient 8), and his mother had FS in childhood. Four patients (patients 15, 18, 30, and 31) were diagnosed with unclassified epilepsy.

### Structural Alterations in the *GABRG2* Protein and Genotype-Phenotype Correlation of *GABRG2* Variants

All *GABRG2* variants, including those in reported and our cohort, were analyzed to explore the mechanism underlying genotype-phenotype correlations. To date, 58 patients with 40 different variants have been reported, including 25 missense, 2 splicing, and 13 destructive variants (7 frameshift, 5 nonsense variants, and 1 small deletion variant). Their clinical characteristics are listed in Table 2. In our cohort of 35 patients, 23 unique variants were identified, including 16 missense variants, 4 splicing variants, 1 nonsense variant, 1 frameshift variant, and 1 small deletion variant. Overall, including our data and those from the literature, we reviewed data from 63 distinct variants identified in 93 unrelated patients (Figure 1 and Tables 1, 2). The variants included 41 missense variants (41/63, 65.1%), eight frameshift variants (8/63, 12.7%), six nonsense variants (6/63, 9.5%), six splicing variants (6/63, 9.5%), and two small deletion variants (2/63, 3.2%).

**TABLE 2:** The genotype and phenotype of 58 patients reported in literature with *GABRG2* variants related to epilepsy.

	Cases	Variants	Seizure onset age	Seizure types	Seizures fever sensitivity: Y or N	EEG	Brain MRI	Developmental	Diagnosis	Other clinical findings
Kang et al., 2019	2	*Inheritance* p.S8R (×2)	22 months 68 months	FS FS	N Y	Not reported	Not reported	Not reported	Genetic epilepsy	Not reported
Huang et al., 2012	1	*de novo* p.Q40X	Not reported	Not reported	NA	Not reported	Not reported	Not reported	Dravet syndrome	Not reported
Ishii et al., 2014	2	*Paternal* p.Q40X (×2)	2 months	Not reported	Y	Not reported	Not reported	Not reported	Dravet syndrome (×2)	Not reported
Shi et al., 2010	1	*de novo* p.N79S	15 years	GTCS	Y	Not reported	Not reported	normal	epilepsy	Not reported
Wallace et al., 2001	1	*de novo* p.R82Q	13 months	Not reported	Not reported	GSS, SW	Not reported	Not reported	GEFS+	Not reported
Stosser et al., 2018	1	*de novo* p.T94K	Not reported	Not reported	Not reported	Not reported	Not reported	Not reported	Epilepsy related neurodevelopmental disorders	Not reported
Lachance-Touchette et al., 2011	1	*de novo* p.P83S	5 months	GTCS	Y	GSS, SW	Not reported	normal	Genetic epilepsy	Not reported
Komulainen-Ebrahim et al., 2019	3	*de novo* p.P282T p.S306F *Paternal* p.P83S	4 years 2 days 9 months	Ats, GTCS, MS Hemi clonic GTCS, AS	Not reported	GSS, SW, FS FS	Cerebral cortical dysplasia Not reported normal	GDD GDD GDD	LGS-like EIMFS LGS	Nystagmus, feeding problems, hypotonia, movement disorders Feeding problems, hypotonia NA
Ma et al., 2019	1	*de novo* p.R136*	Neonatal	GTCS, MS, FS	Not reported	SB	Not reported	GDD	Ohtahara syndrome	Not reported
Johnston et al., 2014	1	*Inheritance* p.R136*	24 months	Not reported	Y	Not reported	Not reported	normal	GEFS +	Not reported
Shen et al., 2017	8	*de novo* p.A106T (×2) p.I107T p.P282S p.R323Q (×2) p.R323W p.F343L	1 day/3 months 1.5 months 1 year 10 months/ 1 year 11 months 1 year	GTCS, TS/TS, GTCS, Ats TS, ES GTCS, AS FS, GTCS, MS, AS/FS, MS, Ats, AAS GTCS, AS TS	Not reported	Normal/DS, MF DS, FSS GSS, MF GSS/GSS GSS DS, FSS	Delayed myelination of white matter/ ventricular enlargement Normal Normal Normal/normal Normal thin corpus callosum	GDD (×8)	EE (×8)	nystagmus, hypotonia/ hypotonia, nystagmus, movement disorders hypotonia, nystagmus, hand stereotype, chorea hypotonia normal/ hypotonia, mild ataxia normal hypotonia
Zou et al., 2017	5	*De novo* p.A106T p.A106T p.A106T p.A106T p.A106T	4 months 3 months 6 weeks 1 day 2 day	GTCS FS FS, GTCS FS, GTCS, MS FS, GTCS, MS	Not reported	GSS, SW SW PSW FSS SW	Normal Enlargement of lateral ventricle and dysplasia of frontotemporal region Normal Normal Normal	GDD (×5)	EOEE (×5)	Ataxia, movement disorders, Visual impairment Ataxia, visual impairment Hypotonia Hypotonia, stereotype Ataxia, visual impairment, nystagmus
Yamamoto et al., 2019	1	*De novo* p.A106T	4 months	ES	Not reported	PSW, MF	Not reported	GDD	EOEE	Dystonia, nystagmus
Epi4K consortium and Epilepsy Phenome/Genome Project, 2017	1	*Inheritance* p.R177P	Not reported	Not reported	Not reported	Not reported	Not reported	normal	Genetic epilepsy	Not reported
Salam et al., 2012	1	*unknown* p.N196N	Not reported	Not reported	Y	normal	Not reported	normal	FS	Not reported
Parrini et al., 2017	1	*De novo* p.Y274C	3 years	GTCS, MS, AtS	Not reported	Not reported	Not reported	delay	EMAS	Not reported
Perucca et al., 2017	1	*Maternal* p.I218S	18 months	AS, GTCS	Not reported	GSS	normal	normal	CAE	Not reported
Boillot et al., 2015	5	*Inheritance* p.E402fs*3 p.R196* p.V462fs*33 p.P59fs*12 p.M199V	24 months 20 months 14 months Not reported 6 months	FS Not reported Not reported GTCS GTCS, AS	Y	FSS NA NA NA NA	normal Not reported Not reported Not reported Not reported	normal	GEFS+	Not reported
May et al., 2018	3	*unknown* p.Y213* c.452_ 455delTCTT c.769-2T > G	Not reported	Not reported	Not reported	Not reported	Not reported	Not reported	Genetic epilepsy	Not reported
Peng et al., 2019	1	*De novo* p.R323W	Not reported	Not reported	Y	Not reported	Not reported	Not reported	Dravet syndrome	Not reported
Baulac et al., 2001	1	*Inheritance* p.K328M	Not reported	Not reported	Y	Not reported	Not reported	Not reported	GEFS +	Not reported
Reinthaler et al., 2015	5	*Inheritance* c.769-2T > G(×2) p.G257R p.R232Q p.I389V	Not reported	Not reported	Not reported	Not reported	Not reported	Not reported	BECT (×5)	Not reported
Hernandez et al., 2017	1	*De novo* p.P302L	1 year	FS, GTCS, AtS, MS	Y	normal	Not reported	delay	Dravet syndrome	NA
Carvill et al., 2013	1	*De novo* p.R323Q	8 months	FS, AS, AtS, MS, GTCS	Not reported	normal	Not reported	normal	EMAS	Not reported
Angione et al., 2019	4	*Paternal* p.R363Q p.K374del *Unknown* c.770-1G > A p. K371T	3 years 2 years 20 months 3 years	GTCS, AtS, TS, MS, AAS GTCS, AS MS, AS, GTCS GTCS, AS, MS, AtS	Not reported	DS, GSS, SW, MF GSS, DS, SW GSS, SW GSS	normal (×4)	GDD GDD normal normal	LGS EMAS EMAS EMAS	ASD NA NA NA
Cogliati et al., 2019	1	*De novo* c.937_938delinsGG	Not reported	Not reported	Not reported	Not reported	Not reported	GDD	Rett syndrome	Ataxia, hand stereotype
Della Mina et al., 2015	1	*Paternal* c.351dupT	1 year	GTCS	Y	Not reported	Not reported	normal	GEFS +	Not reported
Tian et al., 2013	1	*Inheritance* c.1329delC	Not reported	Not reported	Y	Not reported	Not reported	Not reported	GEFS +	Not reported
Harkin et al., 2002	1	*Inheritance* p.Q390*	3 months	FS, GTCS, MS, AtS, SE	Y	GSS, SW	normal	delay	GEFS+	photosensitivity
Kananura et al., 2002	1	*Inheritance* c.769-2T > G	4 years	AS	Not reported	Not reported	Not reported	normal	CAE	Not reported

AAS, atypical absence seizure; ADHD, attention deficit and hyperactivity disorder; AS, absence seizure; ASD, autistic spectrum disorder; AtS, atonic seizure; CAE, childhood absence epilepsy; CS, clonic seizure; DS, diffuse slow waves; EMIFS, epilepsy of infancy with migrating focal seizures; EOEE, early-onset epileptic encephalopathy; GDD, global developmental delay; GSS, generalized seizures discharge; LGS, Lennox-Gastaut syndrome; EE, epileptic encephalopathy; EMAS, epilepsy with myoclonic atonic seizure; ES, epileptic spasms; FS, focal seizure; FSS, focal seizure discharge; FTS, focal tonic seizure; GTCS, generalized tonic-clonic seizure; SE, status epilepticus; MA, myoclonic atonic seizure; TS, tonic seizure; MS, myoclonic seizure; MF, multifocal; EEG, electroencephalography; SB, Suppression-burst; PSW, poly-spike wave; SW, spike wave; NA, not applicable. *Represents the nonsense variant.

Most variants (42/63, 66.7%) were located in the extracellular region and transmembrane region of the protein. Two were located at amino acid position 323 (p.R323Q and p.R323W), representing a potential variant hotspot. The p.R323Q variant was detected in 11 patients (patients 22–30, Carvill et al., 2013; Shen et al., 2017), whereas the p.R323W variant was found in 3 patients (patients 20–21, Peng et al., 2019). In the literature, the phenotypes of some patients with epilepsy carrying *GABRG2* variants were not described in detail; therefore, we were only able to analyze the genotype and phenotype correlations in our cohort. In our cohort, seven recurrent variants are located in the transmembrane region of the channel, including p.P302L, p.A303T, p.T310I, p.T317I, p.R323Q, p.R323W, and p.S346F (Figure 1). Six of them (p.P302L, p.A303T, p.T310I, p.T317I, p.R323Q, and p.R323W) are located in the M2 domain. The p.S346F variant was located in the M3 region. Nine variants were located toward the extracellular region, including p.T90R, p.T90M, p.A106T, p.W121C, p.R125P, p.R125C, p.N140S, p.N167K, and p.P205H. The p.R429X variant is located in the inner part of the cytoplasmic domain. The proportions of patients with variants in the extracellular region and in the transmembrane region who experienced developmental delay were 40% (4/10) and 63.2% (12/19), respectively.

**FIGURE 3:**
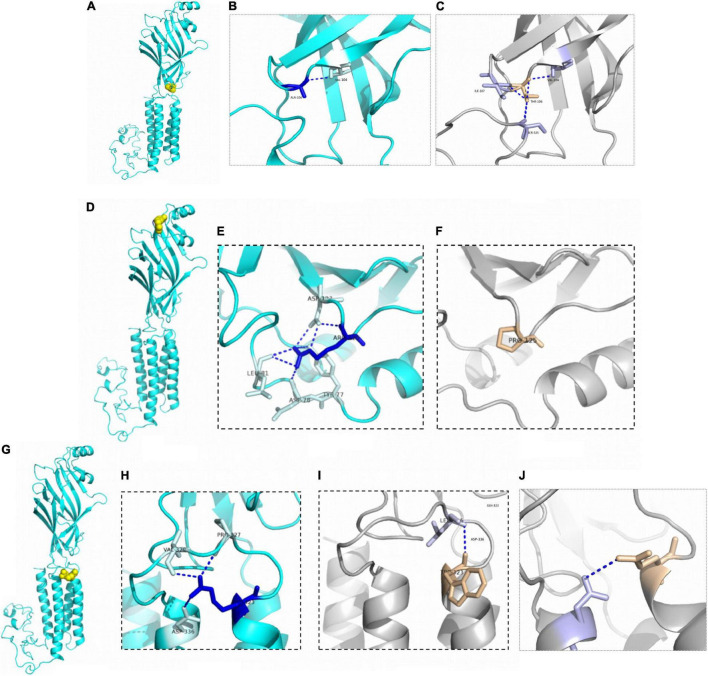
Structural modeling of *GABRG2* variants (A106T, R125P, R323W, and R323Q). **(A)** The position of the 106th amino acid in the subunit (yellow). **(B)** Wild-type 106A forms ionic bonds with surrounding amino acid residues. **(C)** The number of ionic bonds between variant 125T amino acid residues and surrounding amino acid residues is increased. **(D)** The position of the 125th amino acid in the subunit (yellow). **(E)** Wild-type 125R forms ionic bonds with surrounding amino acid residues. **(F)** The number of ionic bonds between variant 125P amino acid residues and surrounding amino acid residues is reduced. **(G)** The position of the 323rd amino acid in the subunit (yellow). **(H)** Wild-type 323R forms ionic bonds with surrounding amino acid residues. **(I)** Change in forces between variant 323W amino acid residues and adjacent α helix. **(J)** Change in forces between variant 323Q amino acid residues.

Patient 3 and patient 4 carried the same *GABRG2* variant (p.A106T) that was located in the extracellular region. Both patients presented with DEE. However, a patient with the R125P variant near the A106T variant was diagnosed with FS+. The R323Q and R323W variants (potential variant hotspots) are located in the transmembrane region. The molecular effect of the missense variants was further analyzed by protein modeling using PyMOL 2.3. Residue A106 originally formed one hydrogen bond with V104. When alanine 106 was replaced by threonine, the hydrogen bonds with I107 and S325 were reestablished. Residue R125 originally formed three hydrogen bonds with D123, two with L81, and one each with Y77 and D78. When arginine 125 was replaced with proline acid, the hydrogen bonds with Y77, D78, L81 and D123 were all destroyed. Residue R323 originally formed one hydrogen bond with residues P327, V329 and D336. In contrast, when arginine 323 was replaced by tryptophan, the hydrogen bonds with residues P327, V329 and D336 were all destroyed, and only one hydrogen bond was retained with L326. Additionally, when arginine 323 was replaced by glutamine, the hydrogen bonds with residues P327, and V329 were destroyed, and only one hydrogen bond was retained with D336 (Figure 3).

### Treatment and Follow-up

Treatment information was available for 35 patients. The final follow-up age ranged from 11 months to 17 years old. At the last follow-up, 25 patients (71.4%, 25/35) were seizure-free for 1 year to 7.5 years. Effective ASMs in terms of seizure freedom included valproate monotherapy (*n* = 9), levetiracetam monotherapy (*n* = 6), perampanel monotherapy (*n* = 1), valproate and levetiracetam combination therapy (*n* = 5), multitherapy (valproate, topiramate and perampanel combination therapy, *n* = 1), and biotherapy other than valproate combine with levetiracetam in 5 patients (patients 2, 3, 4, 12, and 33).

Ten patients still had seizures. One patient (patient 14) was diagnosed with Dravet syndrome. During the last follow-up visit, he still had frequent eyelid myoclonic seizures at the age of 10 years and 6 months. He showed no response to three different ASMs (valproate, levetiracetam, and clobazam). One patient (patient 29) with intractable epilepsy and developmental delay tried three ASMs (valproate, lamotrigine, and oxcarbazepine), but she still experienced cluster focal seizures. When she was treated with perampanel, and the seizure frequency was markedly reduced. Her afebrile seizures were controlled, but she still experienced fever-induced seizures. Patient 16 did not use ASMs due to poor parental compliance, and the seizures were not controlled. For the 7 other patients, the follow-up was too short to evaluate the effect of medication.

## Discussion

Pathogenic *GABRG2* variants have been reported in patients with epilepsy, developmental delays and behavioral disorders. The phenotypic spectrum of *GABRG2* variants extends to the pharmacoresistant epilepsies, including Dravet syndrome and developmental epileptic encephalopathies (DEEs) (Harkin et al., 2002; Shen et al., 2017; Oyrer et al., 2018). Recently, *GABRG2* was included in DEE74 (OMIM: 618396). The pathogenic variants in *GABRG2* (p.Q40X) were first reported in a patient with Dravet syndrome in Kanaumi et al. (2004). In 2013, Carvill (Carvill et al., 2013) reported a male patient with epilepsy presenting myoclonic-atonic seizures (EMAS) who carried a *GABRG2* variant (p.R323Q) and experienced multiple seizure types, including GTCS, absence seizures, atonic seizures, myoclonic seizures, and tonic-clonic seizures. Additionally, Zou et al. (2017) identified 5 patients with seizures carrying the same *GABRG2 de novo* variants (p.A106T) associated with DEE. To date, *GABRG2* variants have been reported in 58 patients who presented with epilepsy in multiple epilepsy centers (Table 2). However, the largest number of patients evaluated in a single study was only 8. To date, systematic research on the phenotypic spectrum and prognosis of *GABRG2* variants related to epilepsy is lacking. Here, we identified 31 novel and 4 previously reported *GABRG2* variants in patients with epilepsy (Sun et al., 2008; Shen et al., 2017; Zou et al., 2017). The phenotypic spectrum and prognosis of patients with *GABRG2*-related epilepsy were further studied.

Among the 35 patients with *GABRG2* variants in our cohort, the seizure onset age in 62.9% of patients (22/35) was during the 1st year of life, and the seizure onset ages of the remaining patients ranged from 1 year and 1 month to 2 years and 10 months. In previous reports, the seizure onset age ranged from birth to 15 years (median age: 1 year) (Table 2). This observation indicated that the seizure onset age of *GABRG2*-related epilepsy occurred mainly before 1 year of age. In our cohort, we identified several key phenotypic features as part of the *GABRG2*-related disease spectrum; 91.4% of patients had fever-sensitive seizures, 68.6% of patients had focal seizures, and most of the patients (71.4%) were seizure-free after ASMs treatment.

Seizures with fever sensitivity have been reported in some patients; however, the percentage of patients presenting this feature had not been delineated. In previous reports, 47.4% (18/38) of patients with *GABRG2* variants experienced seizures associated with fever-sensitivity (Table 2). Fever sensitivity was not reported in the remaining patients. Boillot et al. (2015) reported 5 families with inherited *GABRG2* variants who had febrile seizures and temporal lobe epilepsy. One patient (p.P302L) who experienced febrile seizures, multiple seizure types, and early psychomotor and language developmental delay was diagnosed with Dravet syndrome (Hernandez et al., 2017).

We observed developmental delay in 42.8% (15/35) of patients with *GABRG2* variants, while 66.7% (24/36) of the previously published patients (available data) had developmental delay (Table 2). Most of the patients in our study were still young, and the proportion of patients with developmental delay should be further studied in a large cohort. However, Komulainen-Ebrahim et al. (2019) reported 3 patients with *GABRG2* variants presenting with global developmental delay. Eight patients with *de novo GABRG2* variants associated with epileptic encephalopathies were reported by Shen et al. (2017). In our cohort, the clinical phenotypes of patients with normal development were mostly diagnosed with FS and FS+. The patients with developmental delay mostly had an early seizure onset, and multiple seizure types, some of which were diagnosed with developmental and epileptic encephalopathy.

In our study, no specific EEG pattern in the seizure evolution was observed in the patients with *GABRG2* variants. Interictal EEG performed in patients with epileptic encephalopathy patients, showed epileptiform discharges, including focal and multifocal spike waves, generalized spike waves and polyspike waves. Of the 58 patients reported previously, 51.7% of patients (30/58) were monitored for epileptiform discharges in interictal EEG. One patient with variant p. R136* presented a suppression burst pattern on interictal EEG (Ma et al., 2019). In our cohort, 33.3% (11/33) of patients had a normal EEG, which may be related to the benign phenotype of most children. However, only 13.3% (4/30) of patients had a normal EEG in the published cohort, and the difference may be related to the patients recruited from different research centers; the number of patients must be further expanded for an in-depth analysis. Brain MRI of patients with *GABRG2* variants was usually normal. In our cohort, the brain MRI of 2 patients (patients 3 and 25) was abnormal. These two patients had severe developmental delays. Their phenotypes included DEE, developmental delay and epilepsy. In the published studies, only 22.7% (5/22) of patients had an abnormal MRI: delayed myelination of white matter in one, ventricular enlargement in one, thin corpus callosum in one, and all with epileptic encephalopathy (Shen et al., 2017); enlargement of lateral ventricles and dysplasia of the frontotemporal region in one patient with early-onset epileptic encephalopathy (Zou et al., 2017); and cerebral cortical dysplasia in one patient with an LGS-like syndrome (Komulainen-Ebrahim et al., 2019). All these five patients had a global developmental delay.

Several studies have documented the phenotypic heterogeneity of *GABRG2*-related epilepsy. Of the 58 patients with *GABRG2* variants reported previously, the epilepsy phenotypes were varied (Table 2). The phenotypic spectrum observed for *GABRG2* variants, ranging from febrile seizures to epileptic encephalopathy, is similar to those of the other GABA_*A*_ receptor genes *GABRA1*, *GABRB2*, and *GABRB3* (Johannesen et al., 2016; Moller et al., 2017; Yang et al., 2020, 2021). However, the prognosis of patients with *GABRG2*-related epilepsy is better than that of patients carrying variants in the other three genes. In our cohort, 57.1% (20/35) of patients presented with milder phenotypes, including febrile seizures, febrile seizures plus, focal epilepsy and unclassified epilepsy with normal development. Additionally, 38.1% (16/42) of patients manifested milder phenotypes than those in the previously published cohort (Table 2).

The mechanism of the phenotypic variation caused by GABA_*A*_ receptor family genes is unclear. From the perspective of biological functions, the γ2 subunit (gene *GABRG2*) plays a critical role in GABA_*A*_ receptor trafficking and localization at the postsynapse and yields benzodiazepine-sensitive and Zn^2+^-insensitive GABA_*A*_ receptors (Alldred et al., 2005; Kang and Macdonald, 2016). *GABRG2* functions as a part of GABA_*A*_ receptor complexes, potentially explaining the variability of the clinical phenotypes. Further analysis showed that the *GABRG2* missense variants associated with severe epilepsy phenotypes were mainly clustered in the transmembrane region from the M1 region to the M3 domain. However, the variants located in the extracellular region were associated with phenotypes ranging from mild to severe. Moreover, few variants were located in the cytoplasmic region.

Bioinformatics analysis supported the deleterious effects of the p.R125P and p.R323W variants. The number of ion bonds between amino acid residues decreased in p.R125P according to the structural modeling. These changes may partially be responsible for the instability of protein structure. Arginine is an amino acid that increases the stability of proteins to a certain extent, and the variant at this site may reduce the stability of proteins (Cai et al., 2018). In our cohort, patient 6 with the R125P variant manifested febrile seizures plus. Nine patients were detected with the p.R323Q variant of *GABRG2*. Two patients were detected with the p.R323W variant of *GABRG2.* However, their phenotypes were different. The p.R323W and p.R323Q variants were located in the transmembrane domain. According to cryo-EM, the transmembrane domain is composed of a five-fold symmetric of the α-helix structure (Laverty et al., 2019). Both the R323W and R323Q variants change the ionic bonds with the adjacent α-helix according to structural modeling. Structural studies have shown that the transmembrane domain of the GABA receptor has larger flexibility (Zhu et al., 2018), and the variant in the adjacent α-helix also presents local flexibility in the transmembrane domain in some aspects. This genetic study may provide potential prospects for personalized medicine. The precise mechanism of *GABRG2*-related epilepsy is complex and requires further study in the future. The lack of functional studies limits our understanding of the impact of variants on proteins (loss or gain function).

Valproate and levetiracetam treatment might be suitable for patients harboring *GABRG2* variants. In this study, eight patients were effectively treated with valproate and levetiracetam. Previously, few reports described the effect of ASMs on patients with *GABRG2* variants. Zou reported that seizures were effectively controlled in two patients with *GABRG2* variant (A106T)- related epileptic encephalopathies after treatment with oxcarbazepine (Zou et al., 2017). In our cohort, patient 3 achieved seizure control after receiving treatment with oxcarbazepine. However, oxcarbazepine was ineffective in two patients. One patient with intractable epilepsy had a reduction in seizures after receiving perampanel treatment. Perampanel, is a novel non-competitive α-amino-3-hydroxyl-5-methyl-4-isoxazole-propionate (AMPA) receptor antagonist. An imbalance between glutamate and gamma-aminobutyric acid neurotransmitter systems may lead to hyperexcitability (Engelborghs et al., 2000). However, due to the limited number of patients with follow-up data, further studies are needed in the future. In our previous study of other types of GABA_*A*_ receptor-related epilepsy, we found that patients with *GABRB2* and *GABRB3* variant-related epilepsy patients also had a good response to valproate and levetiracetam (Yang et al., 2020, 2021). The heterogeneity of the clinical presentations related to the *GABRG2* variants makes early diagnosis difficult, and the lack of explanation for this heterogeneity does not currently allow personalized treatment.

## Data Availability Statement

The original contributions presented in the study are included in the article/Supplementary Files, further inquiries can be directed to the corresponding author/s.

## Ethics Statement

The studies involving human participants were reviewed and approved by the Ethics Committee of Peking University First Hospital. Written informed consent to participate in this study was provided by the participants’ legal guardian/next of kin. Written informed consent was obtained from the individual(s), and minor(s)’ legal guardian/next of kin, for the publication of any potentially identifiable images or data included in this article.

## Author Contributions

YY wrote the article under the supervision of YZ. XN, MC, QZ, JD, XT, YW, JY, WS, WW, JM, YL, XY, XZ, TJ, ZY, JL, YS, HZ, SS, DS, and YJ had collected relevant patient information. All authors read and approved the final manuscript.

## Conflict of Interest

The authors declare that the research was conducted in the absence of any commercial or financial relationships that could be construed as a potential conflict of interest.

## Publisher’s Note

All claims expressed in this article are solely those of the authors and do not necessarily represent those of their affiliated organizations, or those of the publisher, the editors and the reviewers. Any product that may be evaluated in this article, or claim that may be made by its manufacturer, is not guaranteed or endorsed by the publisher.

## Footnotes

1
https://gnomad.broadinstitute.org

2
https://www.mutationtaster.org/

3
http://genetics.bwh.harvard.edu/pph2/

4
http://provean.jcvi.org/protein_batch_submit.php?species=human
