# Supplementary material for: Phenotypic Spectrum and Prognosis of Epilepsy Patients With GABRG2 Variants
Source: Front Mol Neurosci. 2022 Mar 14;15:809163. doi: 10.3389/fnmol.2022.809163 (PMC8964129; doi:10.3389/fnmol.2022.809163)
Supplement: Supplementary file 1 [file Table_1.DOCX]

Table S1 Pathogenicity analysis of 35 patients of *GABRG2* variants and ACMG rating（NM_000816）

| # | Variant | Inheritance | PolyPhen-2 | Mutation taster | SIFT | gnomeAD | Classification (ACMG 2017) |
| --- | --- | --- | --- | --- | --- | --- | --- |
| 1 | c.269C>G/p.T90R | *De novo* | Damaging (1) | Damaging (1) | Damaging (0) | / | PS2+PM2_Supporting+PP3 |
| 2 | c.269C>T/p.T90M | Maternal | Damaging (1) | Damaging (1) | Damaging (0) | 0.000008871 | PM2_Supporting+PP3 |
| 3 | c.316G>A/p.A106T | *De novo* | Possibly damaging (0.785) | Damaging (1) | Tolerated (0.545) | / | PS1+PM1+PM2+PM6 |
| 4 | c.316G>A/p.A106T | *De novo* | Possibly damaging (0.785) | Damaging (1) | Tolerated (0.545) | / | PS1+PM1+PM2+PM6 |
| 5 | c.363G>C/p.W121C | *De novo* | Damaging (1) | Damaging (1) | Damaging (0) | / | PS2+PM2_Supporting+PP3 |
| 6 | c.374G>C/p.R125P | Maternal | Damaging (1) | Damaging (1) | Damaging (0.001) | / | PM2_Supporting+PP3 |
| 7 | c.373C>T/p.R125C | Maternal | Damaging (1) | Damaging (1) | Damaging (0) | / | PM2+PP3 |
| 8 | c.419A>G/p.N140S | Maternal | Possibly damaging (0.498) | Damaging (1) | Tolerated (0.44) | / | PM2 |
| 9 | c.501C>A/p.N167K | *De novo* | Damaging (0.996) | Damaging (1) | Damaging (0) | / | PS2+PM2_Supporting+PP3 |
| 10 | c.614C>A/p.P205H | *De novo* | Damaging (1) | Damaging (1) | Damaging (0) | / | PS2+PM2+PP3 |
| 11 | c.631+4A>G | Maternal | / | / | / | / | PM2+PP3 |
| 12 | c.631+5G>T | Paternal | / | / | / | / | PM2-supporting+PP3 |
| 13 | c.922+1G>T | Maternal | / | / | / | / | PVS1+PM2_Supporting |
| 14 | c.905C>T/p.P302L | *De novo* | Possibly damaging (0.713) | Damaging (1) | Damaging (0) | / | PS2+PS3_Supporting+PM2_Supporting+PP3 |
| 15 | c.907G>A/p.A303T | *De novo* | Damaging (1) | Damaging (1) | Damaging (0) | / | PS2+PM2+PP3 |
| 16 | c.917C>T/p.S306F | *De novo* | Damaging (0.998) | Damaging (1) | Damaging (0.012) | / | PS2+PM1+PM2+PM5+PP3 |
| 17 | c.929C>T/p.T310I | *De novo* | Damaging (1) | Damaging (1) | Damaging (0) | / | PS2+PM2_Supporting+PP3 |
| 18 | c.929C>T/p.T310I | *De novo* | Damaging (1) | Damaging (1) | Damaging (0) | / | PS2+PM2_Supporting+PP3 |
| 19 | c.950C>T/p.T317I | *De novo* | Damaging (1) | Damaging (1) | Damaging (0.001) | / | PS2+PM2_Supporting+PP3 |
| 20 | c.967C>T/p.R323W | Maternal | Damaging (1) | Damaging (1) | Damaging (0) | / | PS4+PM2+PM5+PP3 |
| 21 | c.967C>T/p.R323W | *De novo* | Damaging (1) | Damaging (1) | Damaging (0) | / | PS2+PS4+PM1+PM2+PM5+PP3+PP4 |
| 22 | c.968G>A/p.R323Q | Paternal | Damaging (1) | Damaging (1) | Damaging (0) | 0.000003976 | PS1+PM1+PM2+PP3+PP1 |
| 23 | c.968G>A/p.R323Q | *De novo* | Damaging (1) | Damaging (1) | Damaging (0) | 0.000003976 | PS2+PS3+PS4_Moderate+PM2+PP3 |
| 24 | c.968G>A/p.R323Q | *De novo* | Damaging (1) | Damaging (1) | Damaging (0) | 0.000003976 | PS1+PS2+PM2+PP3 |
| 25 | c.968G>A/p.R323Q | *De novo* | Damaging (1) | Damaging (1) | Damaging (0) | 0.000003976 | PS2+PS1+PM1+PM2+PM |
| 26 | c.968G>A/p.R323Q | *De novo* | Damaging (1) | Damaging (1) | Damaging (0) | 0.000003976 | PS2+PS1+PM1+PM2+PM |
| 27 | c.968G>A/p.R323Q | *De novo* | Damaging (1) | Damaging (1) | Damaging (0) | 0.000003976 | PS2+PS1+PM1+PM2+PM |
